# Fakir Journalism

**Published:** 1895-10

**Authors:** 


### FAKIR JOURNALISM.

Under this title the *Tri-State Medical Journal* for July rises to make a few forcible remarks:

"Fakirism exists in journalism to an alarming extent. To the uninitiated the field of medical journalism is an undiscovered country. It is only after one has been on the inside that he can properly discriminate between those medical magazines which are conducted upon an honest basis and those which claim the earth while possessing little of it.

"There are different ways of running and building a medical journal. One way is to resort to exaggeration to deceive the advertiser: 'In less than thirty days our books showed a list of 5,800 paid subscribers,' is the statement made by one journal which went up like a rocket, and is now coming down. Such statements would cause Don Quixote and Munchausen to hide their faces in shame. Yet this is a mild-mannered misstatement compared with some. A certain journal is known to claim a circulation varying from 7,800 to 10,000, and yet never prints more than 1,000 copies, including sample copies and exchanges. And so the tricks resorted to by some might be multiplied indefinitely.

"There is only one correct way to conduct a medical journal. That is to be honest with yourself, honest with your subscribers, and honest with your advertisers. In the course of events this plan will outstrip all others, and will result in the permanent prosperity of the journal and the journalist. One subscriber gained and kept is better for all parties concerned than ten sampled or one hundred claimed by a Munchausen sheet."

With which every one will agree, we think, who is "on the inside" and has the opportunity to observe something of the making and maintaining a good journal. The above remarks might well have been extended to include some thoughts on those publications, the one-fourth medical journal and three-fourths advertising literature of drug houses, proprietary medicine manufacturers, etc.; also something about those journals with full page interleaved advertisements. But perhaps that's another story which we may hear later.

